# Hard X-Ray Microscope With Submicrometer Spatial Resolution

**DOI:** 10.6028/jres.095.044

**Published:** 1990

**Authors:** Masao Kuriyama, Ronald C. Dobbyn, Richard D. Spal, Harold E. Burdette, David R. Black

**Affiliations:** National Institute of Standards and Technology, Gaithersburg, MD 20899

**Keywords:** CCD detectors, fiber reinforced composite, microtomography, multilayer films, x-ray lens, x-ray microscope

## Abstract

A high-resolution hard x-ray microscope is described. This system is capable of detecting line features as small as 0.6 *µ*m in width, and resolving line pairs 1.2-*µ*m wide and 1.2-*µ*m apart. Three types of two-dimensional image detectors are discussed and compared for use with hard x rays in high resolution. Principles of x-ray image magnification are discussed based on x-ray optics and diffraction physics. Examples of applications are shown in microradiography with fiber reinforced composite materials (SiC in Ti_3_Al Nb) and in diffraction imaging (topography) with device patterns on a silicon single crystal. High-resolution tomography has now become a reality.

## 1. Introduction

New engineering has embarked on a revolutionary period leading to new materials tailored from the atomic scale upward to achieve desired functional properties. Materials scientists are now earnestly designing a variety of new configurations of matter in response to industrial desires for sophisticated properties in high-performance applications. Advances in telecommunications and computers, for example, are attributed in part to the successful fabrication of novel microelectronic and photonic device materials designed on the atomic scale. Advances in the structural materials area have also been significant. Rapid solidification, for example, produces metals having excellent combinations of strength, ductility, and corrosion resistance. New processing techniques are quickly adopted to produce improved ceramics and composite materials for sophisticated and demanding applications. Composite materials are composed of dissimilar materials and are highly vulnerable to thermal and mechanical problems during processing.

Materials science that only emphasizes the designing and processing of new materials configurations, although important and necessary, is not quite adequate to advance materials engineering which industry requires to improve productivity and quality. The structure of all materials when formed is often non-uniform locally over regions of the order of a micrometer. Heterogeneity occurring as interdiffusion layers, grain boundaries, phase interfaces, interacting dislocations, local compositional variations, regionally homogeneous strains (residual stresses), and inhomogeneous strains, etc., often alters the performance behavior of materials from their originally designed applications. Thus, successful fabrication of such tailored materials having structures not found in nature depends entirely on structural details and their influence on properties.

Material failures are often attributed, for example, to residual stresses that may have been produced during fabrication processes. Residual stresses can be measured in some cases with limited precision. However, those are normally represented by an average value over the entire specimen or in a rather large volume. Material failure happens at local catastrophic events. Residual stress values statistically averaged over a macroscopic volume have no significance in shedding light on failure events. In microelectronic devices, where different atoms are doped into mutually coherent layers, the thickness and shape of doped layers may change so as to degrade the functional properties originally designed. What we need is a measurement technique to “see” what happens locally and pinpoint local events of significance with high spatial resolution. Once we recognize such local phenomena, we can devise methods to accurately measure physical quantities which are necessary and meaningful in relation to each local event.

This paper describes one such measurement technique using x-ray imaging for detection of microstructural flaws and defects. X-ray beams can penetrate through materials to give two-dimensional images in transmission with high resolution. The current technique described in this paper is capable of detecting lines 0.6-*µ*m wide, and resolving line pairs 1.2-*µ*m wide and 1.2-*µ*m apart [1,2]. Image data are digitized and displayed on a video monitor. An example of applications to fiber-reinforced-metal-matrix composites will demonstrate that the x-ray microscope gives two-dimensional microradiographic images in high resolution for the evaluation of processed materials. An appropriate set of such two-dimensional images can be used to construct a three dimensional image of the material with sufficiently high spatial resolution [3–8]. This is high-resolution x-ray tomography.

Furthermore, examples of applications to microelectronic materials indicate that the x-ray microscope will be a powerful tool, when combined with the diffraction imaging technique, to challenge interface problems from both the scientific and the engineering aspect.

## 2. Area Detectors for X-Ray Imaging

For microtomography, it is essential to obtain a set of two-dimensional images in transmission with high spatial resolution and excellent signal to noise ratio in either microradiography or diffraction imaging (topography). In order to achieve high spatial resolution, a microprobe x-ray beam can be employed, and the transmitted (or diffracted) beam is received by an open-window x-ray photon counter, as either the probe beam or sample is translated in two orthogonal directions to complete a two-dimensional scan. However, the preparation of a microprobe beam requires x-ray focussing, and beam divergence is inevitable. Images from objects in a sample of a finite thickness are, therefore, expanded and overlap with one another, thus deteriorating spatial resolution, and defeating the originally desired high-resolution imaging scheme. Micrometer resolution is unreachable. Besides, it is cumbersome to complete a single two-dimensional image by scanning.

Two-dimensional array detectors offer an efficient way to obtain two-dimensional images. A microprobe beam scan in combination with a two-dimensional detector still suffers the fundamental problem with divergence in producing high resolution images, no matter how small the pixel size of the detector. A parallel beam with a sufficiently large cross section is better suited for two-dimensional imaging with these detectors.

There are three major types of image detectors currently available [9,10]. One is the combination of an image intensifier stage and either a charge-injection-device (CID) type or a charge-coupled-device (CCD) solid state image detector [11]. The intensifier stage consists of a photocathode and a photoelectron multiplier, currently being microchannel plates. X-ray photons must be converted to visible light photons by a phosphor screen that is made of a fine powder or single crystal scintillator at the photocathode. This type of detector is called an indirect image detector. The second type that belongs to a class of direct image detectors is a vidicon detector [12–15], in which a photoconductive target is scanned by an electron beam. X-ray photons change a local electric resistance of the photoconductor giving rise to a different charge separation rate, locally, when the electron beam is rastered. The vidicon detector produces images of reasonable resolution, but is poor in sensitivity at low flux levels of photons. The third is a CCD detector itself, which should belong to the class of direct imaging, although the majority of applications to hard x-ray radiation (1 keV and above) favors the use of phosphors in front of CCD detectors to avoid radiation damage in the detector device.

The CCD is an analog shift register in which an entire sequence of charge packets created by photons can be shifted simultaneously along the surface of a silicon chip by applying clock voltages to overlying insulated gate electrodes. The electric charge varies from one packet to the next. The sequence of charge packets is scanned as they move past an output electrode connected to an on-chip amplifier. In the “frame transfer” type of scientific detectors, the CCD registers are illuminated directly by the (visible or x-ray) light from the imaged scene.

For high spatial resolution, the insertion of phosphors for the conversion of x-ray photons to visible photons is not desirable. X-ray sensitive direct imaging must be used with the CCDs. Direct x-ray bombardment, on the other hand, shortens the lifetime of CCDs due to radiation damage. However, as long as a monochromatic x-ray beam with limited brilliance (≤10^4−5^ photons mm^−2^ s^−1^) is used, the lifetime does not appear to be significantly affected. We have employed a direct imaging method with frame transfer CCDs for several years. The present CCD image detectors in our synchrotron radiation laboratory are described in the following.

The CCD substrate is 50 Ωcm doped Si, which is the diode structure type and is itself a complex integrated circuit. This CCD is designed to perform high-precision *light* (not x-ray) photometry, but can be used in the x-ray photon sensitive mode, that is, the direct conversion of x-ray photons to electric charges. The surface of the detector is illuminated by 5–20-keV x-ray photons. The pixels are arrayed to give the total area of 1.032 × 1.032 cm with 516 × 516 pixels; the pixel size is 20 × 20 *µ*m (smaller pixel sizes may be available commercially). The capacity of each pixel is limited to the total of 250,000 electrons, which is equivalent to about one hundred 8-keV photons, assuming 100% quantum efficiency. The depletion depth is 15 *µ*m. The quantum efficiency is measured to be about 20% at 8-keV photon energy, where charge collection outside the depletion region has been ignored.

Readout is in the frame transfer mode with the noise of less than 25 electrons per pixel, when the detector is thermoelectrically cooled to –40 °C. The dark current is negligible for exposures of several minutes. The readout charges are digitized to 14 bits by an analog to digital converter and stored in computer memory. The readout time is 20 *µ*s per pixel, thus requiring 5 s for full array display. The minimum integration time is currently limited to 0.1 s by the shutter speed, while the maximum integration time is longer than 1000 s, only limited by the dark current level. Digital data are recorded on a hard disk or 1/2″ 9 track magnetic tape and images can be processed on the spot. No significant degradation of image quality was observed during the first 6 months. After more than 1 year of operation, some slight local lightening of images has begun to be noticeable. No serious damage has been observed to affect the lifetime. In the present experience, it is not the number of photons, but the dose rate that seems to influence the increase of the dark current in certain regions of the detector. The performance of the present detector is summarized to be 25 line pairs/mm with less than 10^7^ photons/cm^2^ at 5 to 20 keV energy, being operated only in the photon counting mode.

Although CCD image detectors provide an ideal image data collection method with a sufficiently small pixel size, they are not quite good enough for high-resolution imaging. Various efforts to make the pixel size smaller, e.g., less than 1 *µ*m, are expected to face tremendous technical problems. However, the spatial resolution, limited by the pixel size, can be improved by the direct image magnification technique which is equivalent to an optical lens capable of magnifying images [16,17]. The combination of a CCD detector and an x-ray image magnifier circumvents the current technical problems associated with the reduction of pixel size in CCD detectors and makes it possible to create an x-ray microscope with less than 1-*µ*m resolution [18].

## 3. Imaging Principles of Magnification in Two Dimensions

**Figure 1:**
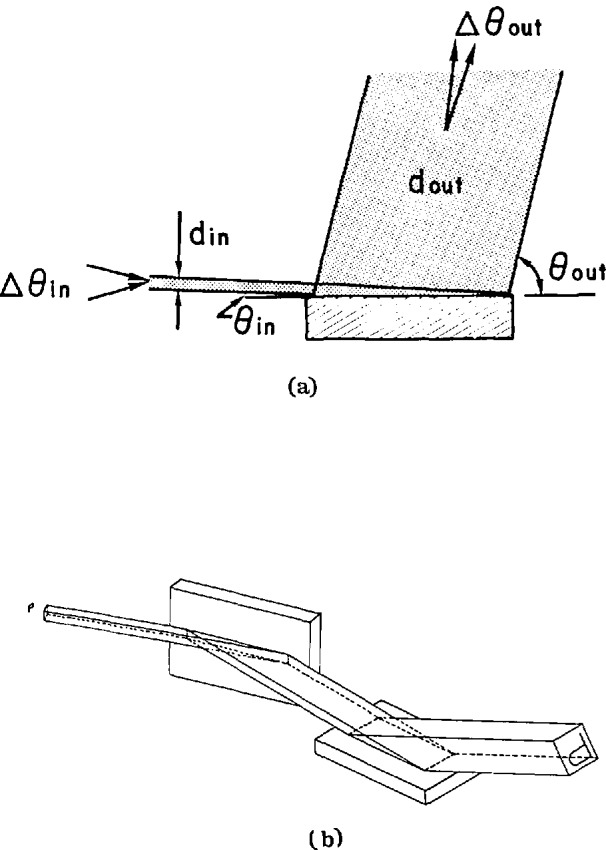
(a) Asymmetric Bragg diffraction showing one-dimensional magnification of an x-ray beam. The cross section of the incoming beam *d*_in_ is magnified by a factor *m* to produce the outgoing beam *d*_out_, while the parallelism of the outgoing beam Δ*θ*_out_ becomes more improved by the same factor *m* than the incoming beam Δ*θ*_in_ (b) use of two asymmetric diffractions to obtain a two-dimensional (normal) magnification of images contained in an x-ray beam. Notice how the image “P” is magnified and inverted.

When a monochromatic x-ray beam is diffracted from the surface of a highly perfect crystal and the diffracting planes in use are not parallel to the crystal surface, the diffraction is termed asymmetric. There are three aspects of asymmetric diffraction which are important for image magnification—beam magnification, reflectivity, and the beam acceptance angle of the crystal for the diffraction [16]. Figure 1(a) shows schematically the asymmetric diffraction geometry. An incident beam of x rays is magnified in one dimension (in the plane of diffraction) by a factor *m* given by

$$m=\sin\;\theta_{\mathrm{out}}/\sin\;\theta_{\mathrm{in}},$$ (1)

where *θ*_in_ and *θ*_out_ are the angles between the crystal surface and the incoming and outgoing beams, respectively. Obviously high magnifications are obtained when *θ*_in_ is very small. Alternatively the magnification factor can be written in terms of the Bragg angle for the diffraction *θ*_B_ and the angle α (cut angle) between the diffracting plane and the crystal surface as

$$m=\sin\;\left(\theta_{B}+\alpha \right)/\sin\;\left(\theta_{B}-\alpha \right).$$ (2)

Typically, Si (111) or (220) is used for the diffraction with various cut angle α’s to provide appropriate ranges of magnification for different energies (wavelengths) of x rays. The practical levels of magnification range from about 10 to 200.

Dynamical diffraction effects that take place in perfect crystals are crucial to understanding the reason why such a geometrical principle leads to a working “magnifying lens” for x rays. In dynamical diffraction, a parallel beam of monochromatic x rays which strikes a crystal with an angle slightly off the Bragg condition can experience diffraction. The ratio of the diffracted total flux (photons/s) to the incident total flux is called the reflectivity and is a function of the deviation from the Bragg condition. This function is called the rocking curve. For a thick perfect crystal with no absorption, this ratio is unity for a range of angles centered about the Bragg condition (called the rocking curve width or range of reflection ω), and falls to zero rapidly for larger deviation from the Bragg condition. Thus, this angular range of reflection gives the beam acceptance angle of the crystal for the diffraction. Because the reflectivity is unity, regardless of the diffracting plane, the intensity (or brilliance) (photons/s·cm^2^) of a parallel beam magnified in one dimension by asymmetric diffraction from a perfect crystal is decreased by a factor *m*^−1^ only because of the magnification of the beam area.

In dynamical diffraction from a perfect crystal, an incoming parallel beam is diffracted into a parallel beam. If the incident beam deviates from the Bragg condition by an angle ϕ, then the diffracted beam deviates from the Bragg diffracted direction by an angle *m*^−1^ϕ, since the Bragg law is “loosened” to restrict photon momentum conservation only in two dimensions, unlike the Bragg law for kinematical scattering which is equivalent to the photon momentum conservation law in three dimensions [19,20]. In reality, the incident x-ray beam has an angular divergence Δ*θ*_in_ due mostly to the source size of x rays. For imaging, it is the source size rather than the beam divergence that is important. At a single point on the magnifying crystal, the source size is given by

$$\Delta \theta_{\mathrm{in}}=H/L,$$ (3)

where *H* is the actual source height and *L* is the distance between the source and the single observation point. For *H* = 150 *μ*m and *L* = 15 m, Δ*θ*_in_ is about 2 arcsec. This source size can be made effectively smaller by inserting monochromator or x-ray optical systems in the optical path before the observation point. In such a case, the smaller apparent source size Δ*θ*_in_, instead of eq (3), should be used in the following discussions.

Hence from the two-dimensional Bragg law, the total divergence of outgoing beams, Δ*θ*_out_, becomes

$$\Delta \theta_{\mathrm{out}}=m^{-1}\Delta \theta_{\mathrm{in}}.$$ (4)

The higher the magnification, the more parallel becomes the outgoing beam. However, only those rays accepted by the crystal within the angular range ω are diffracted with unity reflectivity. Rays striking the crystal outside the range ω are essentially not diffracted. Therefore, if *Δθ*_in_ <ω,

$$\Delta \theta_{\mathrm{out}}=m^{-1}\Delta \theta_{\mathrm{in}},$$ (5)

and if Δθ_in_≥ω,

$$\Delta \theta_{\mathrm{out}}=m^{-1}\omega =m^{-1/2}\omega_{s},$$ (6)

where the acceptance angle (the rocking curve width) for asymmetrical diffraction is given by [21]

$$\omega =m^{1/2}\omega_{s},$$ (7)

Here, ω_s_ is the acceptance angle for a symmetric diffraction for the same diffracting plane. For the 111 diffraction from Si with 8-keV radiation where ω_s_ is ~5 arcsec (1 arcsec = π/648 000rad), the maximum limit of Δ*θ*_out_ is 1 arcsec for the case of magnification of 25, and Δ*θ*_out_ can be smaller, if the incident beam is prepared to be less than 25 arcsec in divergence. The latter condition can easily be achieved with an appropriate monochromator system at synchrotron beam lines, discussed below.

The small value of Δ*θ*_out_ guarantees the one-to-one correspondence of magnified images and the unmagnified images. This is an essential factor for making a “lens.” In the case of a divergent incident beam, the intensity (brilliance) of the diffracted beam is usually called the integrated intensity because it arises from contributions over the entire acceptance angle region. The integrated intensity is proportional to ω and hence is proportional to the x-ray structure factor of a given magnifying crystal. However, if the divergence of the beam is less than ω as in the case of eq (5), the diffracted total flux (photons/s) is equal to the incident total flux and the diffracted intensity (photons/s cm^2^) is decreased by only the geometrical factor *m*^−1^.

Radiation from special x-ray sources, such as a synchrotron, has a powerful continuous energy spectral distribution—polychromatic (white) radiation. A “monochromatic” beam is normally prepared from such white radiation for a specific photon energy by an appropriate monochromator system. Since this beam is different in quality from the usual characteristic radiation, the effect of the radiation source size should be evaluated. The source size is defined as an angle subtended to view (through any additional x-ray optical system, such as a monochromator) the entire source from single points on the magnifying crystal. This angle now gives Δ*θ*_in_ for the “monochromatic” beam prepared from white radiation. It should be noted that there is an almost uniform distribution of x-ray energies in ray trajectories within Δ*θ*_in_. As in the case of the purely (say, characteristic) monochromatic beam discussed above, dynamical diffraction effects determine the energy pass-band for each ray trajectory in accordance with the acceptance angle range ω at a single point on the magnifying crystal. The average photon energy is, to be exact, different at individual single points due to the Bragg law.

The photon energy spread from one extreme edge of the beam to the other extreme is, for example, 40 eV using Si 111 asymmetric diffraction for a magnification of 25, assuming ~40 arcsec as the maximum vertical divergence in the synchrotron radiation beam without the insertion of a monochromator. However, the insertion of the monochromator system (or the magnifying crystal) which utilizes asymmetric diffraction in the magnification mode reduces this divergence to a much smaller value, because the effective beam height viewed by such an asymmetric cut crystal (say, 5×5 cm in size) for the diffraction is the length projected perpendicular to the beam due to the small grazing angle (say, 1°) which is about 1 mm and equivalent to Δ*θ*_in_=10 arcsec. The energy spread in actual use is thus determined by the two extreme edges of the monochromator crystal in the magnification mode and ultimately by the extreme two edges of the magnifying crystal. It should be noted, however, that this energy spread is evaluated from the beam *divergence*, not the source size.

On the other hand, the pass-band Δ*E*_in_ at the observation point is determined by the smallest of the pass-bands of crystals acting as x-ray optical elements in the optical path. This pass-band is described by the same ω as defined before for such a crystal, and is given by

$$\Delta E_{\mathrm{in}}/E=\omega \;\cot\;\theta_{B},$$ (8)

where *θ*_B_ is the Bragg angle for the x rays impinging on the observation point with the average energy *E.* For Si 111 diffraction with *m* =25 using the 8-keV monochromatization, eq (8) gives Δ*E*_in_= 3 eV with ω ~ 25 arcsec. Hence, even in a single ray trajectory of the beam path, the energy dispersion given by eq (8) is inevitable for the “monochromatic” beam, because the beam in this trajectory contains a continuous energy spectrum. Since Δ*E*_out_=Δ*E*_in_ in elastic scattering, Δ*θ*_out_ is not affected. For the “monochromatic” beam, spatial resolution (particularly in diffraction imaging) is, therefore, affected by the pass-band as well as the source size Δ*θ*_in_. The dispersive arrangement of the monochromator system is required to reduce the pass-band.

If the x-ray energy of “monochromatic” beams can be tuned to any desired value, it is easily seen from eq (1) that the magnification factor of the same magnifying crystal can be varied at will, thus providing an image-zooming capability which is quite useful as described later.

For two-dimensional imaging, two one-dimensional magnifying crystals are arranged in two orthogonal directions to obtain an undistorted image of the sample which is placed in front of the magnifying crystals [16,22]. As illustrated in figure 1(b), detailed structures of the sample in the beam, shown as a little P, are now magnified, shown as a large P, and received by a CCD image detector. To guarantee undistorted images, care must be taken with the mutual alignment of these crystals. The plane of diffraction, defined as the plane containing the incoming beam and the normal to the diffracting plane (and also the outgoing beam) for each of the two successive diffractions must be orthogonal. Hence the first diffraction magnifies the beam horizontally and the second in a perpendicular direction. The distance between the sample and the “lenses” and between each lens must be minimized.

Since the reciprocity theorem holds for x rays, this system compresses the image in the demagnification mode. This mode of operation can provide very small, intense beams for other applications, including x-ray lithography.

## 4. A Hard X-Ray Microscope

**Figure 2:**
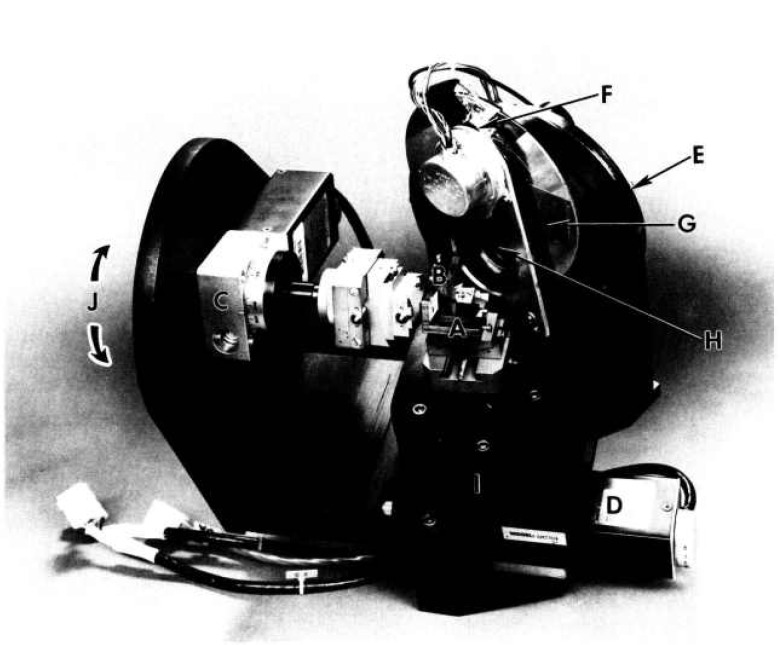
The high-resolution hard x-ray microscope capable of detecting submicrometer features. A and B are asymmetrically-cut x-ray diffraction optics controlled by microstepping rotators C and D. E is an x-ray sensitive CCD array. The carrousel mounted on the CCD camera head holds a photodiode F, a lead foil shutter G and a clear-line-of-sight aperture H. I is the rotator for the camera assembly and J is another rotator (not shown) that mounts the entire microscope assembly.

The combination of the x-ray sensitive CCD detector and the x-ray image magnification technique now produces a hard x-ray microscope capable of resolving micrometer features [18]. A photograph of such a microscope is shown in figure 2. Asymmetrically-cut x-ray optical elements, A and B, are orthogonally aligned to function as the objective lenses of the microscope. Control of these optical elements is provided by their respective rotators, C and D, which are step motors driven with a step size of 0.9 arcsec. The beam containing the object image impinges, via the objective lenses, on an x-ray sensitive CCD array, E, which has been described previously. The carrousel’ mounted on the CCD camera head, E, holds a PIN (photo) diode, F, for alignment of the optical elements, A and B. The x-ray sensitive area of the PIN diode is 4 × 4 mm and is completely light-tight. Also mounted on the carrousel are a lead foil (0.15-cm thick) shutter, G, to protect the CCD camera, and a clear-line-of-sight aperture, H, to receive the image on the camera. The carrousel is controlled by the main computer which controls beam optics including a monochromator system. A shutter for the image exposure is separately set in front of the sample and is controlled by the CCD camera computer. The assembly of the camera head and the carrousel is mounted on a rotator, I, which rotates at various speeds with 60 arcsec/step. The entire microscope assembly is mounted on a rotator J (not shown), which is located on the left side of this figure. This rotator is similar to I. I and J are controlled by the main computer for beam optics.

The microscope is aligned for operation, using the main computer and the readout from the PIN diode, F. Scan-and-search-peak routines are applied to the first lens A and the second B in sequence by rotating the angular position of the PIN diode in appropriate scattering angles. During the operation, slits which are located in the monochromator system are set just inside the magnified image of the incident beam. After a series of routine operations are completed, the CCD area detector is rotated in place of the PIN diode. The refinement of the angles of A and B follows under the observation of the image on a monitor screen of the CCD detector to “tweak up” the microscope system.

For the orthogonality alignment of A and B, the importance of which was discussed earlier, insert a gold 25×25 *μ*m grid mesh in the sample holder, and adjust the setting of B with respect to A, using an auxiliary dc motor connected to one of the arcs of the goniometer head that holds B, while viewing the two-dimensional image on the CCD monitor screen. Rows and columns of the mesh image must become perpendicular to each other. Obviously, when the magnification factor of the microscope is increased by increasing the x-ray energy, the orthogonality must be refined.

For the x-ray microscope, a parallel monochromatic incident beam is highly desirable. Such a beam can be prepared by an asymmetric-cut monochromator crystal with characteristic radiation [11], and by a monochromator system consisting of a crystal, functioning as a prism, and another crystal, functioning as a monochromator, with synchrotron radiation [2,23]. These crystals can be prepared for symmetrical and/or asymmetrical diffraction. In the present use, these crystals are aligned in the non-dispersive mode (note a comment in a previous section). Preliminary calculations and experiments have indicated that the asymmetric (*m*) and asymmetric (1/*m*) arrangement for these crystals gives a better condition for photon flux, but the symmetric (*m* = l) and symmetric arrangement is superior with respect to spatial resolution.

**Figure 3:**
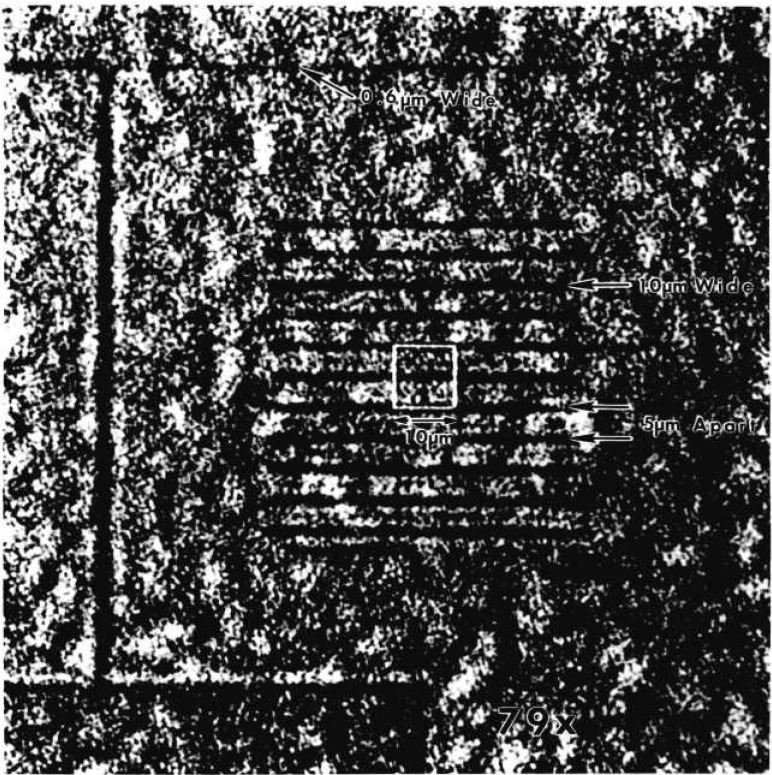
A radiographic image of a pattern of Pd lines on Si. This image was obtained with a magnification of 79 and a wavelength of 1.0 Å (12.3 keV). At this magnification, one CCD pixel represents 0.25 *μ*m.

**Figure 4:**
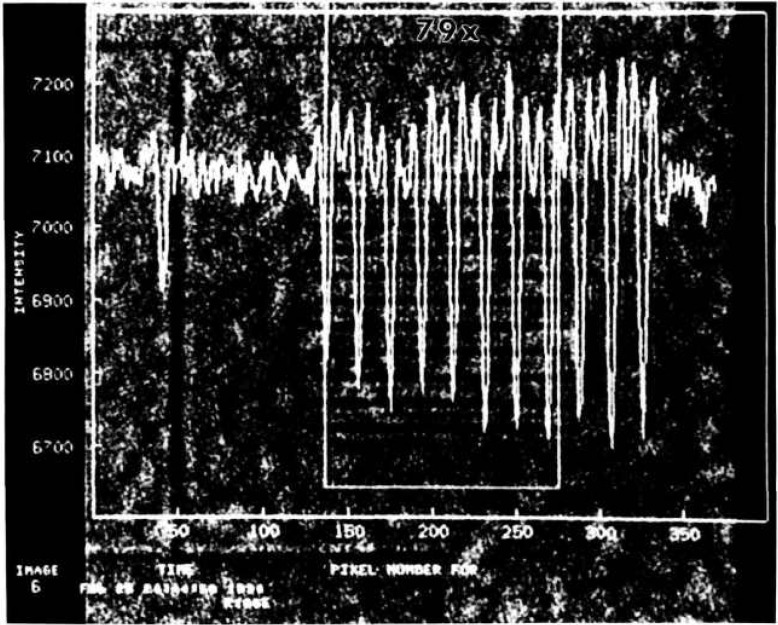
A plot of the average intensity. The average pixel intensity for each horizontal row of pixels is plotted, superimposed on the view seen in figure 3.

Figure 3 shows what has been achieved with the x-ray microscope to date. This is a microradiographic image of a pattern of 0.15-*μ*m thick Pd lines, of various widths, deposited on a 0.38-mm-thick Si wafer. The image was obtained at a magnification of 79 and a wavelength of 1.0 Å (12.25 keV). At this magnification, one CCD pixel represents 0.25 *μ*m. The 11 evenly spaced lines are 1-μm wide and 50-*μ*m long, with a center-to-center spacing of 5 *μ*m. The single line above the 11 lines is 0.6 *μ*m wide. The submicrometer feature is clearly visible for the first time. The image in figure 3 has been normalized to a blank field to correct for intensity variations due to blemishes in the magnifier crystals and non-uniform illumination. This is another advantage of CCD cameras which store digital image data for further image processing and quantitative analyses. Figure 4 shows a plot of the average pixel intensity for each (horizontal) row of pixels, superimposed on the view of figure 4. The dips in intensity for the 11 1-*μ*m lines have an average full-width half maximum (FWHM) of 1.4 *μ*m, while the dip (shown on the left) for the 0.6-*μ*m line has a FWHM of 1.0 *μ*m, in good agreement with the predictions of geometrical optics. However, the secondary dip between each 1-μm primary dip cannot be explained by geometrical optics, and are, at present, attributed to Fresnel diffraction.

**Figure 5:**
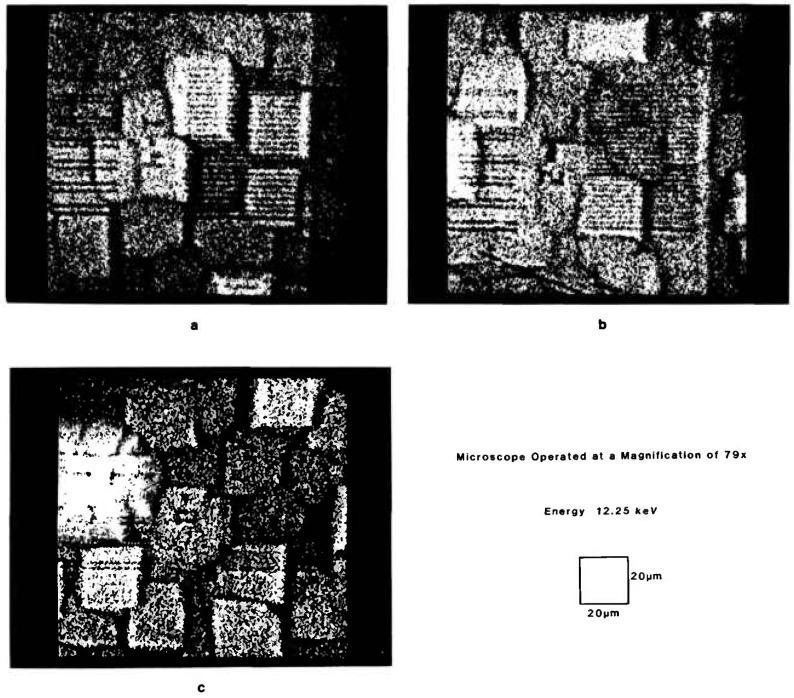
Images of various line pair patterns with the microscope operated at a magnification of 79 × at 12.25 keV, (a) 1.4-*μ*m wide and 1.4-*μ*m apart, (b) 1.2-*μ*m wide and 1.2-*μ*m apart, and (c) 1.0-*μ*m wide and 1.0-μm apart.

For the evaluation of spatial resolution, line pairs of various widths and spacings should be used. Figure 5 (a), (b), and (c) show the images taken from Pd line pair patterns with the microscope operated at a magnification of 79 × at 12.25 keV. The images of line pairs, (a) 1.4-*μ*m wide and 1.4-*μ*m apart and (b) 1.2-μm wide and 1.2-*μ*m apart, clearly show well-resolved patterns, while the image of line pairs, (c) 1.0-*μ*m wide and 1.0-*μ*m apart, is hardly discernible. Although the microscope is able to show isolated submicrometer features as evidenced in figures 3 and 4, the resolution of the microscope should be considered to be 1.2-*μ*m. It should be noted that the Si substrates on which Pd line pair patterns were prepared show many features of microstructure and may have degraded the crispness of the patterns and their images particularly when the features become less than 1 *μ*m.

**Figure 6:**
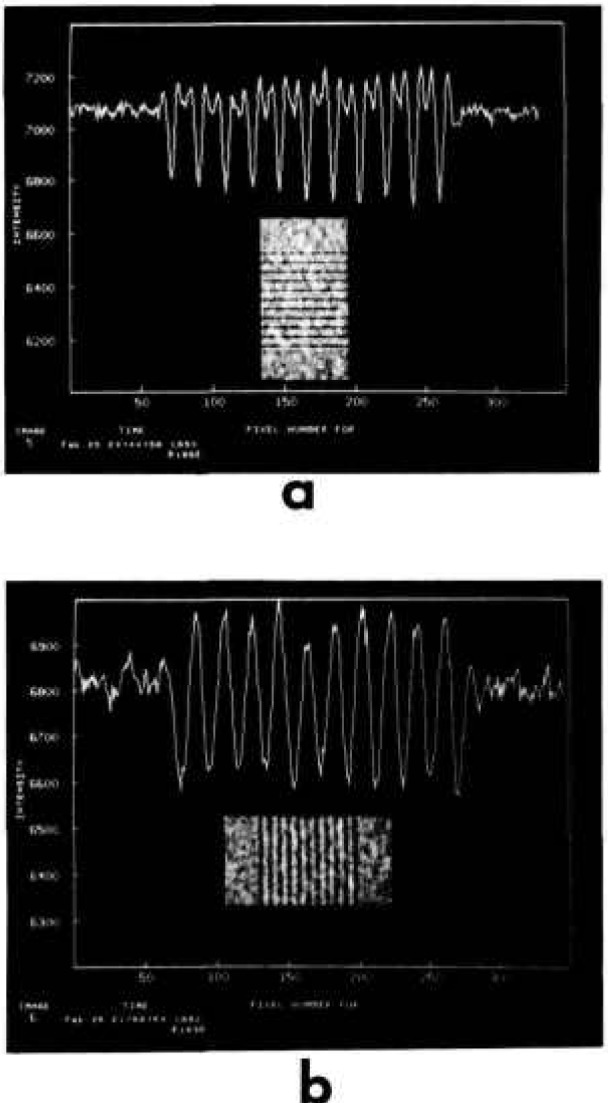
The effect of the vertical and horizontal source sizes. Images were obtained at a magnification of 79 × and 12.25 keV, (a) the line pattern is oriented horizontally for the vertical source size effect, (b) oriented vertically for the horizontal source size effect.

The synchrotron beam line that was used in this work has not yet been equipped with a monochromator system to control the horizontal source size. In this beam line, the horizontal source size of the synchrotron radiation is twice as much as the vertical source size. To evaluate the source size effect, the Pd pattern used in figure 4 was placed in two orthogonal orientations. The pattern object was placed 4 mm from the edge of the first lens A. Figure 6 (a) and (b) show the images taken at a magnification of 79 ×, (a) the line pattern being placed horizontally to detect the vertical source size effect and (b) being placed vertically for the horizontal source size effect. Also shown are the plots of the average pixel intensity for each horizontal and vertical row of pixels, respectively. The broadening or blurring of image (b) is easily recognizable, indicating that the width of each line image is twice as much as those observed in figure 6 a in agreement with the prediction based on the source size of the synchrotron radiation in use.

## 5. Applications

In the microradiographic mode, the hard radiation microscope has many applications in the detection of flaws and defects in all sorts of materials including advanced structural and functional material components, dental, medical and biotechnological materials and in the development of new processing and chemical treatments for these materials, as examples in the dental research area indicate [24,25].

**Figure 7:**
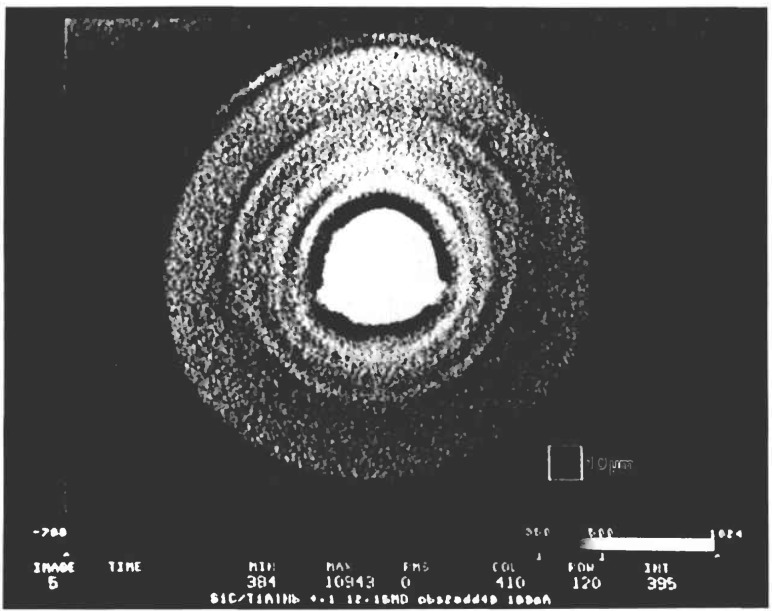
An image of a single SiC fiber in a reinforced Ti_3_Al Nb metal matrix composite at a magnification of 55× and 12.15 keV. The thickness of the sample is about 1.7 mm. The diameter of the fiber is about 130 *μ*m. A graphite core is at the center.

An example of straightforward applications to microradiography is the evaluation of processed materials. Here a reinforced Ti_3_Al Nb metal matrix composite material is used to image a single SiC fiber with the microscope operated at a magnification of 55 × and 12.15 keV. The thickness of the sample is about 1.7 mm. The x-ray beam is transmitted through the sample in a direction parallel to the fiber axis. Figure 7 shows a microradiograph of a single fiber, roughly 130 *μ*m in diameter, surrounded by a Ti_3_Al Nb matrix. A graphite core is at the center. The detailed structure of SiC appears as a bundle composed of layers with great clarity; no pixel images are registered discernibly. The CCD pixel size is equivalent to 0.45 *μ*m under this magnification.

**Figure 8:**
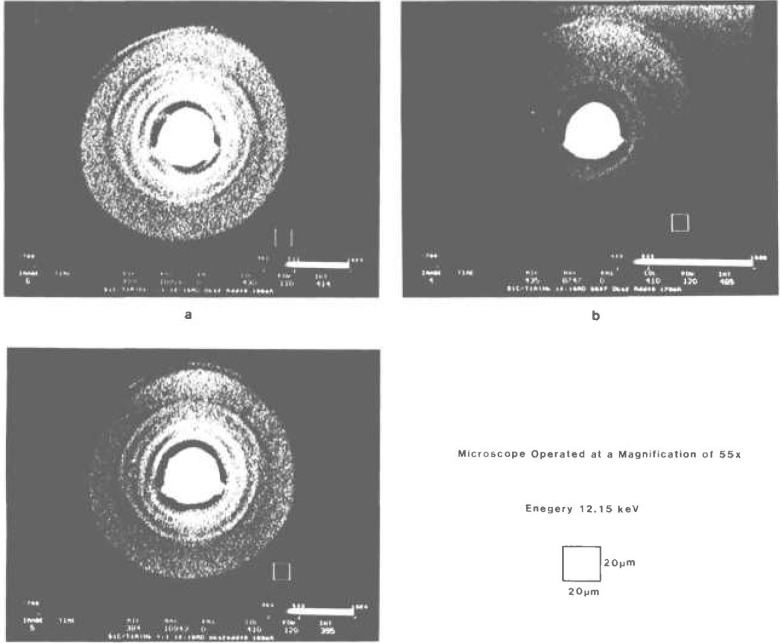
Three views of the SiC fiber at a magnification of 55× and 12.15 keV. The sample is oriented (a) −0.1°, (b) 0.0°, and (c) +0.1° off from the x-ray beam direction.

As in the operation of an optical microscope, the zooming capability of the x-ray microscope is indispensable to first locate such a small object in a wide field of view, using a lower magnification, and then to increase a magnification of the microscope to desired levels. Also necessary is the capability to manipulate the sample orientation while viewing images. In the present application to a single fiber, it is difficult to determine when the x-ray beam is really parallel to the fiber. Figure 8 (a), (b), and (c) show three different views of the fiber with an increment of 0.1° inclined from the x-ray beam. Each shows slightly different aspects of the SiC layers.

**Figure 9:**
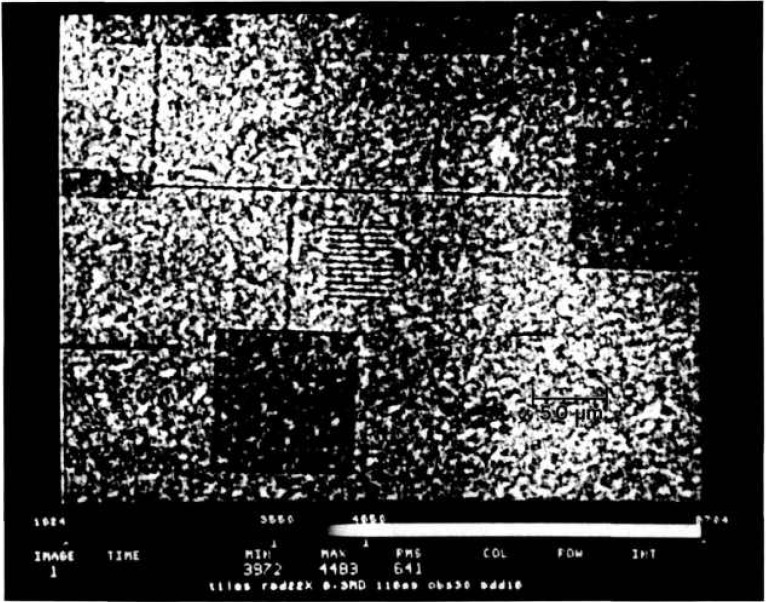
A radiographic image of another section of Pd pattern with a magnification of 22 × at 8.3 keV.

The application of this microscope is not limited to microradiography. Device features used in microelectronics and photonics are prepared by a sequence of depositions of a few atoms at a time and doping in appropriate time intervals. These features are almost perfectly lattice-matched to each other and to the substrate. It is difficult to create images with good contrast from mutually coherent features consisting of similar materials. The physical shape of these features, if visible in microradiography, may not give clues for the understanding of phenomena actually taking place. For example [2], figure 9 shows a microradiographic image with 22 × magnification at 8.3 keV of the Pd pattern shown in figure 3. This shows the physical shape of object features. Since Pd is more absorbing than the Si substrate, contrast is good.

**Figure 10:**
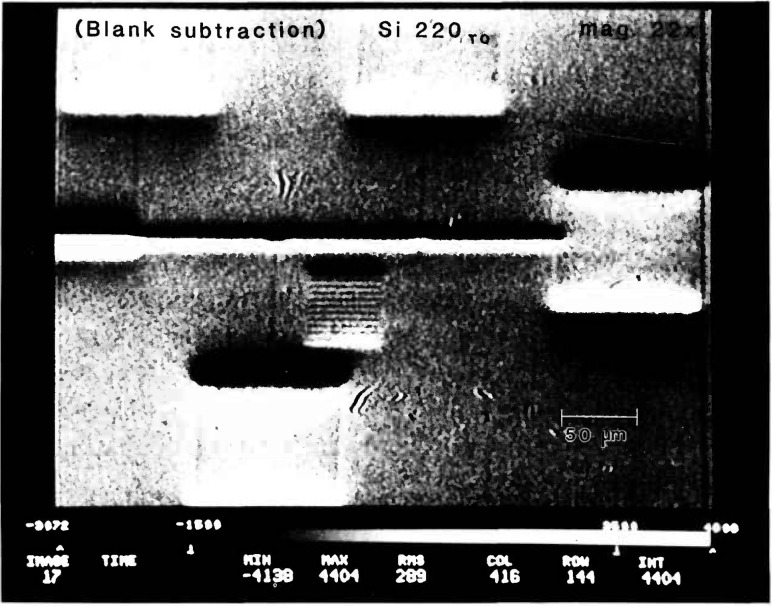
The O-beam image of the Pd pattern for the 220 diffraction in transmission with the same magnification of the microscope as the companion radiographic image in figure 9.

Because this substrate is a single crystal and the Pd pattern is epitaxially coherent it can be brought into a Bragg condition. In transmission two diffracted images appear [1]: one in the forward direction of the incident beam is called the O-beam image, and the other in the Bragg diffracted direction is called the H-beam image. Without moving the microscope from the position set for microradiography, the O-beam image can be viewed on the monitor screen merely by rotating the sample. Figure 10 shows the O-beam image taken in transmission for the 220 diffraction with the microscope set for the same magnification, 22 × at 8.3 keV, as figure 9. In dynamical diffraction, a strain field acts as a scattering center and produces a black and white image [1]. The black/white contrast is expected to be inverted in the H-beam image. This contrast inversion has been confirmed by moving the microscope to view the H-beam image. Therefore, there are interfacial strains around the features. The direction of the atomic shifts in these strained areas can be analyzed in detail.

The ability to reveal strain features in diffraction imaging along with micrographic views of the object can now be utilized to a full extent not only in a qualitative but in a quantitative way to study interface problems. The above example with the Pd pattern opens up an opportunity to challenge the problems associated with advanced microelectronic devices, as described in a previous paper [18]. Since these materials are often highly perfect single crystals, x rays can pass through more than one-half millimeter thick samples, almost regardless of x-ray energy, when the sample is set for Bragg diffraction due to the anomalous transmission or Borrmann effect [1,26,27]. In this mode of imaging—phase contrast microscopy, the important feature is that those transmitted images in diffraction represent true transverse cross sectional images of the layers [1,18].

As demonstrated so far, the hard x-ray microscope is indeed capable of producing two-dimensional images with 1 *μ*m or less spatial resolution in microradiography and diffraction imaging. A set of two-dimensional images is easily collected as a function of rotational angle, as shown in figure 8. A tomographic image can be reconstructed from this set of high-resolution two-dimensional images. To reconstruct a three-dimensional image with 1-μm spatial resolution, the increment of the sample rotational angle is, for example, roughly 0.8° for a cylindrical sample 100 *μ*m in radius. Obviously, the larger the sample, the smaller the required angular increment. The rotator currently in use can easily provide this capability with a well-fixed rotational center controlled by a set of submicrometer translators. Algorithms are available for tomographic reconstruction. A working microtomographic system capable of reaching 1 *μ*m or less spatial resolution has become a reality with the hard radiation microscope described in this paper.
